# COVID-19 and Gut Injury

**DOI:** 10.3390/nu14204409

**Published:** 2022-10-20

**Authors:** Sj Shen, Muxue Gong, Gang Wang, Kamal Dua, Jincheng Xu, Xiaoyue Xu, Gang Liu

**Affiliations:** 1 UNSW Microbiome Research Centre, St George and Sutherland Clinical Campus, University of New South Wales, Sydney, NSW 2217, Australia; 2 School of Clinical Medicine, Bengbu Medicine College, Bengbu 233030, China; 3 Department of Respiratory and Critical Care Medicine, Clinical Research Center for Respiratory Disease, West China Hospital, Sichuan University, Chengdu 610041, China; 4 Discipline of Pharmacy, Graduate School of Health, University of Technology Sydney, Ultimo, NSW 2007, Australia; 5 Faculty of Health, Australian Research Centre in Complementary and Integrative Medicine, University of Technology Sydney, Ultimo, NSW 2007, Australia; 6 Stomatology Department, The First Affiliated Hospital of Bengbu Medical College, Bengbu 233004, China; 7 School of Dental Medicine, Bengbu Medical College, Bengbu 233030, China; 8 School of Population Health, University of New South Wales, Sydney, NSW 2052, Australia; 9 School of Life Sciences, Faculty of Science, University of Technology Sydney, Ultimo, NSW 2007, Australia; 10 Centre for Inflammation, Centenary Institute, Camperdown, NSW 2050, Australia

**Keywords:** COVID-19, gut, microbiota, IBD, ACE2, probiotics

## Abstract

COVID-19 induced by severe acute respiratory syndrome coronavirus 2 (SARS-CoV-2) is currently a pandemic and it has led to more than 620 million patients with 6.56 million deaths globally. Males are more susceptible to COVID-19 infection and associated with a higher chance to develop severe COVID-19 than females. Aged people are at a high risk of COVID-19 infection, while young children have also increased cases. COVID-19 patients typically develop respiratory system pathologies, however symptoms in the gastrointestinal (GI) tract are also very common. Inflammatory cell recruitments and their secreted cytokines are found in the GI tract in COVID-19 patients. Microbiota changes are the key feature in COVID-19 patients with gut injury. Here, we review all current known mechanisms of COVID-19-induced gut injury, and the most acceptable one is that SARS-CoV-2 binds to angiotensin-converting enzyme 2 (ACE2) receptor on host cells in the GI tract. Interestingly, inflammatory bowel disease (IBD) is an inflammatory disorder, but the patients with IBD do not have the increased risk to develop COVID-19. There is currently no cure for COVID-19, but anti-viruses and monoclonal antibodies reduce viral load and shorten the recovery time of the disease. We summarize current therapeutics that target symptoms in the GI tract, including probiotics, ACE2 inhibitors and nutrients. These are promising therapeutic options for COVID-19-induced gut injury.

## 1. Introduction 

Coronavirus disease-2019 (COVID-19) is induced by severe acute respiratory syndrome coronavirus 2 (SARS-CoV-2) that was firstly reported in Wuhan in December 2019. The outbreak of COVID-19 was raised as a big concern by the World Health Organization and it eventually became a pandemic since early 2020. The virus has changed over the past three years, with some strains listed as variants of concern that pose an increased risk to global public health. These variants include Alpha (earliest samples were reported in UK in September 2020), Beta (South Africa, May 2020), Gamma (Brazil, November 2020) Delta (India, October 2020) and most recently Omicron (multiple countries in November 2021). COVID-19 Omicron variants lead to mild symptoms, but it has the fastest and highest infection rate compared with other variants. Recent Omicron BA.4 and BA.5 sub-variants have become the dominate virial strains globally. This virus infection results in respiratory disorders, with COVID-19 patients exhibiting cough, fever, fatigue and some patients developing acute respiratory distress syndrome (ARDS), including lung fibrosis and severe pneumonia [1,2]. COVID-19 also leads to dysregulation in the gastrointestinal (GI) tract and abdominal symptoms in COVID-19 patients, including anorexia, nausea, vomiting and diarrhea. Anal swab specimens from COVID-19 patients tested positive for SARS-CoV-2 nuclei acid and SARS-CoV-2 can be isolated from stool samples from COVID-19 patients [3]. Many researchers have since focused on investigating the link between COVID-19 and GI tract injury, and the mechanisms that underpin the connections of the lung and GI tract. There has been significant progress in understanding of the relationship between the digestive system and SARS-CoV-2 infection. In this review, we will summarize the key pathological features, mechanisms of pathogenesis and treatments of COVID-19-induced GI tract injury. 

## 2. Epidemiology

By October 2022, more than 621 million people have been diagnosed with COVID-19; this includes 247 million in Europe, 175 million in Americas, 82 million in the Western Pacific, 60 million in South-East Asia, 22 million in the Eastern Mediterranean and 9 million in Africa. COVID-19 leads to 6.56 million deaths globally [4]. Patients with COVID-19 show various disorders in the GI tract, with the most commonly reported symptoms being diarrhea, vomiting, nausea and abdominal pain. In an early meta-analysis published in 2020, 7.7% of the included cohorts experienced diarrhea, 7.8% experienced nausea and vomiting, and 3.6% experienced abdominal pain [5]. Interestingly, compared to studies carried out within China, studies from other countries reported a higher proportion of patients with these symptoms [5]. Subsequent reviews reiterated this large variance in patients with GI tract symptoms that cohorts are from numerous countries including China, USA, Europe, Australia and Singapore [6]. GI tract symptoms have since been consistently reported in patients with COVID-19, regardless of whether the patients showed respiratory tract symptoms or not. However, even within the Chinese studies, there are large variations, with the range of patients having diarrhea being from 0% to 36.6% [5,6]. This suggests that apart from overarching geographical and lifestyle factors that may result in deviations at a country level, genetics and other intrinsic factors may play important roles in eliciting GI tract damage in COVID-19 patients.

### 2.1. Sex Differences in COVID-19

Significant sex differences on COVID-19 prevalence have been widely reported. A recent meta-analysis of including more than 220,000 participants shows that the prevalence of COVID-19 is slightly higher in males than females (55% vs. 45%) [7]. Studies with larger cohorts also show that males have greater COVID-19 severity associated with an increased level of inflammation than women [8]. The mortality from COVID-19 in males is 1.3–1.8-fold higher than females [9]. Although it remains unclear why males are more susceptible to COVID-19 infection than females, a possible reason has been proposed that females have a generally stronger immune system than males [8].

### 2.2. Age Difference in COVID-19

Many studies reported that people aged above 65 have a higher chance to acquire COVID-19 infection and develop severe illness from COVID-19. Therefore, this group of people has been recommended as the priority group for vaccination in most countries [10]. However, recent studies show that young children under 5 years of age also have increased risk of getting COVID-19 [11]. Additionally, GI tract symptoms were not only reported in adults, but also in young children. Several studies have shown that diarrhea was presented in 8.8–44.1% and vomiting in 6.4–67.7% of pediatric patients with COVID-19 [11,12,13], and 27 of 171 (15.8%) of a cohort of pediatric patients presenting with asymptomatic respiratory infections [11].

Another complication of GI disease observed in children is multisystem inflammatory syndrome in children (MIS-C). The symptoms of MIS-C include abdominal pain, diarrhea, ongoing fever, cardiac dysfunction and multiple organ failure [14]. Severe MIS-C phenotype and worse prognosis are commonly associated with symptoms including those of the GI tract [15]. Due to similarities in presentation between MIS-C and Kawasaki disease in patients, a recent study has examined and compared the levels of serum nitrogen terminal pro-brain natriuretic peptide (NT-proBNP) and C-reactive protein (CRP) between these patients. They also found that the patients with MIS-C have significantly high levels of NT-proBNP and CRP that result in hyperactivation of the immune response and cytokine storm in these patients [16].

## 3. Clinical Features of COVID-19

**Figure 1:**
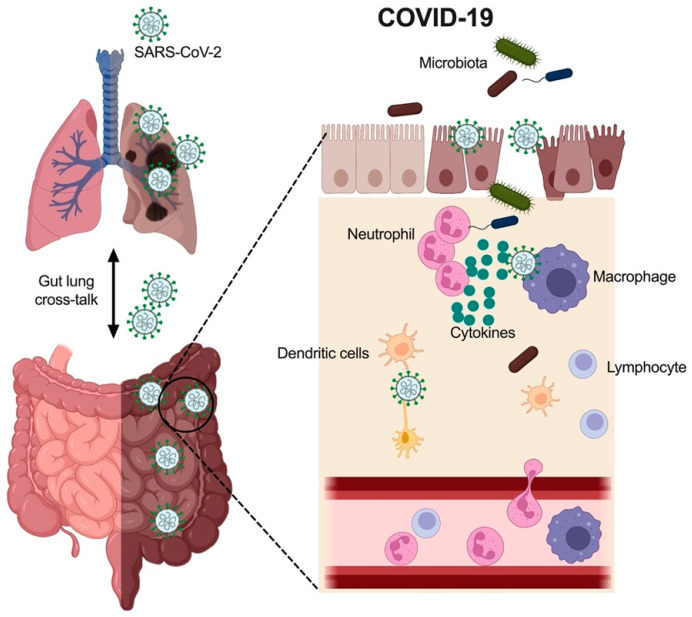
Clinical features of COVID-19-induced gut injury. SARS-CoV-2 infects respiratory system, and the virus translocate to gastrointestinal (GI) tract. This leads to immune cell recruitments in the gut, including neutrophils, macrophages, dendritic cells and lymphocytes. The infection also results in these immune cells to secrete cytokines and microbiota changes in the GI tract.

The GI tract is composed of three main compartments in relation to the immune landscape: the epithelial (mucosal) layer, lamina propria (submucosa), and mucosa/gut-associated lymphoid tissue (MALT/GALT). SARS-CoV-2 infection of the GI epithelium induces an inflammatory and anti-viral response, which has been supported by in vitro experiments using gut organoids [17]. Detection of viral single-stranded RNA (ssRNA) and components by toll-like receptors (TLRs, a group of receptor proteins in innate immune system) elicits the production and release of cytokines, of which the most-studied are interferons (IFNs) and tumor necrosis factor (TNF)-α [18]. In addition, the microbial communities that reside on mucosal surfaces such as within the GI tract (the gut microbiota) have intricate links with host homeostasis and should not be discounted in COVID-19-mediated GI symptoms [19,20,21]. Indeed, COVID-19 alters the gut microbiota (dysbiosis) and induces inflammation and dysregulated immune responses in the gut [22]. Together, these inflammatory mediators promote the recruitment of immune cells that initiate an inflammatory response, leading to tissue damage and GI symptoms (Figure 1).

### 3.1. Immune Cells in COVID-19

One of the initial responses of an infection is the activation of innate immune cells, and COVID-19 is no exception. In particular, resident GI immune cells have the capacity to immediately respond to infiltrating viruses. Studies show that SARS-CoV-2 infection leads to disruption of intestinal epithelial cell barriers and activates immune cells in the GI tract [23]. Additionally, an abnormal gut epithelial–immune interaction drives the inflammatory response in severe COVID-19 patients. However, the exact effect of intestinal inflammation and the role of different type of immune cells in COVID-19 are still unclear. Neutrophils are the first-line immune cells to be recruited to the site of infection. Macrophages and dendritic cells (DCs) that locate the area are normally important in maintaining an appropriate level of regulated immune response that maintains homeostasis between the host and the microbial communities that reside on mucosal surfaces in the GI tract (the gut microbiota) [24]. These cells have a unique capacity to sense disruptions to gut homeostasis. Upon sensing of viral (and other microbial) infections, resident immune cells rapidly produce cytokines and chemokines that initiate the anti-viral responses [25]. ssRNA from SARS-CoV-2 detected by TLR4, TLR7 and TLR8 on antigen-presenting cells such as macrophages quickly elicits the production of pro-inflammatory cytokines including interleukin (IL)-1β, IL-2, IL-8, IFN and TNF-α [26]. These and other molecules initiate the recruitment of cells from the circulation. Both neutrophils and macrophages utilize their phagocytic capability to target viruses and to remove the gut of cell debris, with apoptosis of infected cells contributing to the anti-viral response [27]. Indeed, patients with COVID-19 exhibit neutrophilia with increased levels of fecal calprotectin (a molecule mainly found in neutrophils), indicating the link of neutrophil migration in the GI tract during COVID-19 [28,29]. Patients with severe COVID-19 show significantly higher levels of neutrophil extracellular traps (NETs), circulating double stranded DNA (dsDNA), histone–DNA complexes, neutrophilic elastase, and myeloperoxidase (MPO)–DNA complexes [30]. In addition, stimulation of isolated neutrophils from healthy people with lipopolysaccharide (LPS) in combination with or without β-glucan elicits the production of NETs and increases pro-inflammatory cytokines, including TNF-α, IL-6 and NFκB [30]. Together, these finding suggest neutrophil activity with severe COVID-19.

In addition, DCs can also phagocytose virus and present viral proteins to innate lymphocytes such as natural killer (NK) and NK T cells, or to adaptive lymphocytes such as T and B cells. Plasmacytoid DCs are important producers of type I IFNs [31] and in murine coronavirus infection models (including SARS-CoV-2), type I IFNs are crucial in early stages to facilitate the activation of the adaptive immune response [32,33]. Nevertheless, other coronaviruses, such as Middle-Eastern respiratory syndrome (MERS) and SARS, impair the production of anti-viral cytokines [34], and recent studies have indicated similar impairments of DC function and production of type I IFNs, in addition to decreased cell numbers after SARS-CoV-2 infection [32,35].

Effective induction of the adaptive immune response, and indeed having robust T and B cell responses, are postulated to be important for the resolution of SARS-CoV-2 infection [36]. CD4 T cells aid in generating an effective antibody response in B cells, while also producing anti-viral cytokines, while CD8 T cells can directly kill infected host cells. Indeed, individuals with IL-2-secreting T cells were resistant to infection [37]. T and B cell responses have also been confirmed to be important in SARS-CoV-2 infections in experimental models of COVID-19 in animals [38,39].

In patients with COVID-19, lymphopenia, including decreased CD4 and CD8 T cells, B cells, and NK cells, has been observed, although the breadth of immune cells that SARS-CoV-2 impacts is not consistent across all individuals [40,41,42]. It is currently thought that lymphopenia occurs with direct lysis of lymphocytes, atrophy of lymphoid tissue, or dysregulation of lymphocyte survival and apoptosis [42]. Since inflammation is also observed in the gut, circulating leukocytes could instead be extravasating and accumulating in the GI tissue, but this remains to be elucidated. Nevertheless, some studies suggest that this imbalance in immune homeostasis especially in patients with severe COVID-19 has been linked to GI symptoms such as diarrhea [43].

### 3.2. Cytokines in the Gut with COVID-19

Cytokines link infections with an anti-viral immune response, and also bridge the innate and adaptive arms of the immune response [44]. IFNs are a family of cytokines with potent antiviral properties and includes type I (most predominantly IFN-α and IFN-β), type II (IFN-γ) and type III (IFN-λ) [45]. One effect of IFNs is to prime the neighboring cells for antiviral immune responses. Upon sensing of viral components, IFNs from infected cells bind to IFN receptors (e.g., IFN alpha receptor (IFNAR)1, INFAR2, or IFN lambda receptor (IFNLR1)/IL-10RB) in both an autocrine and paracrine manner [45]. This activates the Janus kinase (JAK)/signal transducer and activator of transcription (STAT) signaling pathway and for IFN regulatory factors to bind IFN-stimulated response elements [46]. Ultimately, this induces the expression of IFN-stimulated genes that are involved in inhibiting viral replication and limiting transmission to other cells [46]. Some mechanisms of IFNs interfering with virus replication include IFNs inducing (1) RNases that degrade viral nucleotides, (2) enzymes such as protein kinase RNA-activated (PKR) that block the translation of viral RNA, and tripartite motif (TRIM) prevent the release of viral particles, and (3) apoptosis of infect cells through upregulation of Fas ligand, programmed death-ligand 1 (PD-L1) and TNF-related apoptosis-inducing ligand (TRAIL) [47]. In addition, IFNs are also involved in the activation of both the innate and adaptive immune responses, such as through the mobilization of cytotoxic T cells for cytolysis of infected cells [48]. Like all battles in nature, there exists an arms race between host antiviral response and viral escape mechanisms. Many viruses including influenza viruses, vaccinia virus, adenovirus, and hepatitis C virus have the capacity to inhibit IFN response in one or multiple methods. These pathways have been extensively reviewed [49], and include molecules that: (1) sequester viral nucleic acid to prevent activation of signaling, (2) reduce the production of IFNs, such as through inhibition of NF-κB, (3) bind IFNs to prevent its binding with and activation of IFN receptors, (4) compete for binding with or inhibit phosphorylation and activation of co-factors such as PKR, which are involved in the IFN signaling pathway. In parallel with these findings on the importance of IFNs in viral infections, earlier studies indicate beneficial functions of IFNs in combating SARS-CoV-2 [50]. However, overactivation of IFN signaling can potentiate damaging inflammatory events instead [51]. Recent studies demonstrate that IFNs have harmful effects on the immune system during COVID-19 [52]. Nevertheless, the exact contribution of IFNs to COVID-19 and especially in how to combat SARS-CoV-2 infection in the gut remains to be fully understood.

Another important anti-viral cytokine is TNF-α. One capacity of TNF-α is the induction of apoptosis in cells [53]. Studies utilizing animals with genetic mutations in the TNF pathway observed increased susceptibility to infection, and cell culture of lung epithelial cells with influenza virus showed a dose-dependent response in the production of TNF-α [54]. Similarly, there is evidence for an important role of TNF-α in COVID-19. A recent study indicated that as COVID-19 progresses into later stages, there is a shift from IFN dominance to TNF-α [55]. In addition, TNF-α secreted by CD4 T cells also correlated with more robust antibody responses, as measured at day 28 post-COVID-19 infection [55]. In patients with various chronic inflammatory conditions that receive anti-TNF therapy, there are lower antibody response to COVID-19 vaccination [56,57], although the same trend was not observed in patients with psoriatic arthritis [58]. There is little evidence on whether or not anti-TNF-mediated inhibition of the humoral response impacts the gut and COVID-19-induced GI damage. TNF-α has been shown to play an important role in balancing cell survival and death, activation of pro-inflammatory NFκB signaling, and gut homeostasis [59]. Depending on the microenvironment, TNF-α has roles in both protection and damage to the gut barrier. Findings from experimental colitis models suggest a role of TNF-α that is protective during acute disease, and deleterious in chronic inflammation [60,61]. Therefore, it is possible that TNF-α has an important anti-viral contribution against SARS-CoV-2, though further research is needed on how the gut microbiota and microenvironment affects signaling pathways and antibody production that are downstream of TNF-α activation.

### 3.3. Gut Microbiota in COVID-19

COVID-19 patients develop alterations to the gut microbiota and metabolite compositions. Patients with severe to critical COVID-19 showed significantly increased microbiota differences both at baseline and after recovery from COVID-19 [62]. Furthermore, there were significantly decreased levels of acetic acid, butyric acid, valeric acid, valproic acid, and isoleucine in severe/critical COVID-19 patients, and all these metabolites except acetic acid remained low even after recovery from disease [62]. Dysbiosis has also been associated with respiratory dysfunction three months following severe COVID-19 [63], whereas a stable gut microbiota composition correlated with favorable COVID-19 progression [64]. Another study found reduced gut microbiota richness in COVID-19 patients six months following infection, and this was associated with increased levels of serum C reactive protein (CRP, an inflammation marker to indicate infection) and severity of disease [65]. Pediatric patients with COVID-19 also showed reduced α- and β-diversity of the gut microbiota composition when compared to healthy controls, with an increase of *Faecalibacterium*, *Fusobacterium*, and *Neisseria*, and reduced abundance of strains including *Bifidobacterium*, *Blautia*, *Ruminococcus*, and *Akkermansia* [66]. Although no pediatric patient developed severe COVID-19, those who were classified as mild or moderate exhibited reduced α-diversity when compared with asymptomatic individuals [66]. Together, these studies indicate that SARS-CoV-2 infection induces dysbiosis that likely adds to the complex immune–microbial interactions in the gut microenvironment to result in GI symptoms.

In addition, the gut environment is also altered in patients with post-acute COVID-19 syndrome (PACS). A recent study reports that patients with PACS have persistent changes in gut microbiota, in addition to GI symptoms including diarrhea (around 5% of study cohort), abdominal pain (around 4%), and nausea (around 3%) at either 3 months or 6 months post-COVID-19 [67].

## 4. Mechanisms Underlying COVID-19 Induced GI Tract Injury

**Figure 2:**
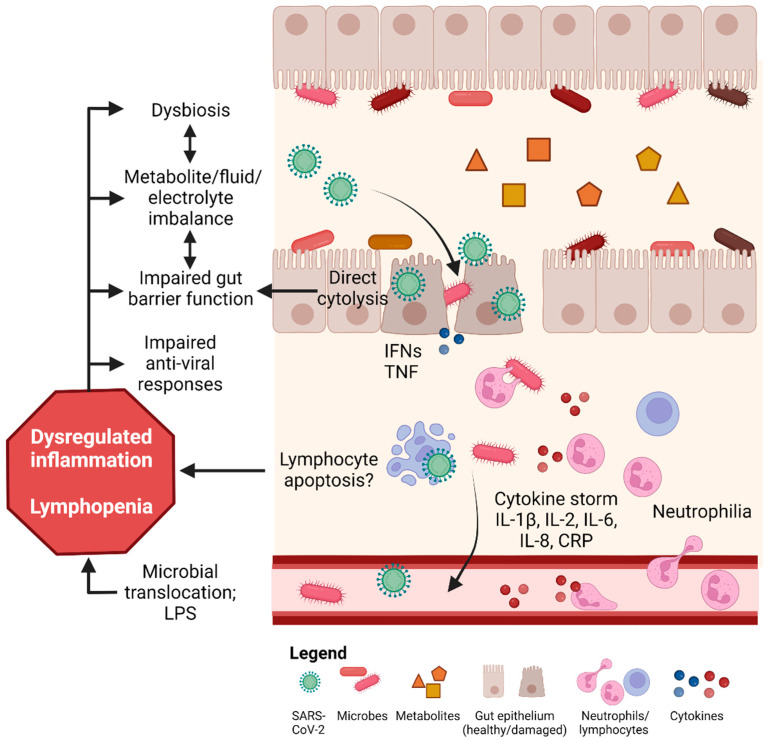
Mechanisms of SARS-CoV-2-induced gut injury. SARS-CoV-2 directly infects gut epithelial cells, and results in impaired gut barrier function and microbial translocation. This induces inflammation (e.g., by cytokine storm) that potentiates dysregulation of gut microbiota, metabolites, electrolytes, and gut barrier functions. The infection of SARS-CoV-2 into the host cells is associated with neutrophilia and lymphopenia, to impairs the host anti-viral immune response that generates a positive feedback loop to promote further infection and damage. These process leads to damage of the GI tract and associated symptoms such as diarrhea. IFNs: interferons. IL: interleukin. CRP: C-reactive protein. LPS: lipopolysaccharide. TNF: tumour necrosis factor.

The SARS-CoV-2 virus binds to the angiotensin-converting enzyme 2 (ACE2) receptor to cause COVID-19 [68]. In the respiratory tract, interaction of the ACE2 receptor on type 2 alveolar cells with SARS-CoV-2 results in pulmonary infection and inflammation. In addition to the respiratory tract, researchers quickly identified ACE2 receptor expression throughout the GI tract, including in the stomach, duodenum, ileum, and colon [69]. Current evidence indicates that SARS-CoV-2 enters cells within the GI tract to disrupt ACE2 receptor expression and initiate inflammation and injury, while also able to modify the gut microbiota and metabolite homeostasis (Figure 2).

### 4.1. SARS-CoV-2 Infection via ACE2 Binding and Injury in the Gut

In the GI tract, the ACE2 receptor is abundantly expressed on epithelial cells in the small and large intestines, and less abundantly expressed in the esophagus [70]. Although SARS-CoV-2 can bind other receptors, little is understood on whether entry into epithelial cells of the GI tract is mediated by binding of receptors other than the ACE2 receptor. After binding, the receptor/virus complex is internalized, and proteases such as transmembrane serine protease 2 cleave the spike protein and enable release of the viral particle into the cells [71]. Evidence indicates that during intestinal infection by SARS-CoV-2, there is direct damage to the epithelial cells, leading to disruptions to the tight junctions and impairment of gut barrier [70,71]. This process ultimately results in dysregulation of fluid balance and GI tract symptoms, such as diarrhea.

During the cell invasion process, there is removal of ACE2 receptor from the cell surface, thus decreasing ACE2 receptor expression. This results in an imbalance in the renin–angiotensin system (RAS) that modulates blood pressure and reduces ACE2-mediated protection against hypertension, which may lead to dysregulated functioning of multiple organ systems including the cardiovascular, pulmonary, and renal systems [72]. However, ACE2 functions at a local GI tract level have only recently been extensively studied. Some roles of ACE2 in the gut includes the regulation of absorption and release of fluids and electrolytes, and regulation of metabolites (glucose and amino acids) [73,74]. At a physiological level, ACE2 mediates the function of the GI tract including blood flow, gut motility, and inflammation [75]. Thus, disruptions to these systems especially in fluid and metabolite homeostasis likely contributes to GI tract symptoms. In addition, these changes in the GI tract are intricately linked to the functions of the gut microbiota, and a result of SARS-CoV-2-mediated dysregulation of ACE2 expression is the downstream dysbiosis. Studies using ACE2-deficient animals observed reduced levels of tryptophan [74,76]. Although not entirely elucidated, changes in tryptophan levels or its sensing consequent in reduced levels of anti-microbial peptides in the gut are likely due to a subset of tryptophan-rich antimicrobial peptides [77], which may explain the observed dysbiosis in the ACE2-deficient animals. In particular, dysbiosis in ACE2-deficient animals was reverted with the supplementation of tryptophan [76], suggesting that nutrient imbalance could be a mechanism of ACE2-mediated dysbiosis.

### 4.2. Dysbiosis and Metabolite Imbalance Induce Gut Inflammation and Injury

Several changes to the gut microbial community have been observed in patients with severe COVID-19, including increased *Bifidobacterium longum*, *Blautia* sp., *Bacteroides nordii* and *Burkholderia* contaminans [78]. Dysbiosis is a known factor associated with impaired gut barrier function and increased permeability [79]. It is unclear exactly how changes in bacterial colonies result in a breakdown of the gut barrier. An overgrowth of bacteria adds stress to the gut epithelial cells [80], while different bacterial ligands (such as LPS from different bacterial genera) differentially modulate immune cell responses and inflammation. Circulating LPS and increased pro-inflammatory cytokine levels correlate with a breakdown in gut barrier integrity [81]. Many studies use endotoxemia, which has been observed in COVID-19 patients as an indirect measure of gut permeability [30].

Dysbiosis also affects the homeostatic balance of metabolites in the gut lumen. COVID-19 patients show alterations in gut microbial communities including decreased abundance of *Faecalibacterium prausnitzii* and reduced capacity for the gut microbiota to produce short-chain fatty acids (SCFAs) such as acetate, propionate, butyrate, valerate and L-isoleucine [62]. While butyrate can be metabolized directly by cells in the gut and helps in maintaining gut barrier integrity and function, all SCFAs have been shown to exert anti-inflammatory and immunomodulatory roles in the host [82]. Interestingly, certain SCFAs such as butyrate and isoleucine were observed to remain at a decreased level even after the COVID-19 patient has recovered [62]. This suggests the long-term impacts of COVID-19 on the gut microbiome and metabolome, potentially inducing prolonged dysregulation of host physiology and immunology. Dysbiosis-induced gut barrier dysfunction, bacterial translocation and inflammation are correlated with COVID-19 and disease severity [78], but also contribute to GI symptoms in these patients.

There is also evidence to indicate malnutrition as a result of SARS-CoV-2 infection [83]. Admission to the intensive care unit has been associated with the thickening of the small intestine [84], which could be an inflammatory response to GI viral infection. In addition, there appears to be less metabolism and absorption of nutrients as determined by metabolomics of feces in patients with COVID-19 [85]. A subset of patients also exhibited more than 5% weight loss, which correlated with increased systemic inflammation and a longer duration of COVID-19 [86]. Together with aforementioned alternations in ACE2 receptor expression and dysbiosis, it is plausible that SARS-CoV-2 infection of epithelial cells in the gut, and especially in the small intestine, results in malnutrition and potentiates dysbiosis, impairment of gut barrier function and systemic inflammation.

Ultimately, this creates a positive feedback loop for increased translocation of gut microbes into systemic circulation, and the potentiation of inflammation [87], culminating in systemic inflammation and cytokine storm that contribute to the worsening of gut damage and increased severity of COVID-19 [87]. Increased circulating levels of bacterial DNA have been observed in patients with severe COVID-19 [88]. A possible reason is an overwhelming and dysregulated response targeting translocated bacteria that is evidenced by overall increased inflammation (IL-6, IL-8, CRP) and reduced immune cells (lymphocytes, CD3, CD4 and CD8 T cells) [78], as well as dysbiosis in the gut. Circulating bacterial components LPS and β-glucan both induce strong immune responses and cytokine release and have been correlated with disease in COVID-19 patients [30]. Increased levels of β-glucan were seen in COVID-19 patients who also exhibited higher levels of TNF-α, IL-1β, IL-6, IL-8, and LPS [30]. It is interesting that the level of β-glucan was not different in COVID-19 patients with mild, moderate, or severe disease, but the levels of TNF-α, IL-8, and LPS significantly increased with increasing disease severity [30]. This could indicate that LPS rather than β-glucan has more direct influence on the cytokine storm. This further reiterates the impact of SARS-CoV-2 infection in the gut, leading to impaired gut permeability and bacterial translocation and potentiates a damaging inflammatory response in the gut.

### 4.3. Other Mechanisms of COVID-19 Induced Gut Injury

Although we have focused on the bacterial and metabolic aspects of COVID-19 induced gut injury, there are other mechanisms that contribute to COVID-19 induced gut injury. For example, recent studies show that CD4 T cells and CXCR3-mediated recruitment of T cells into the gut potentially result in diarrhea associated with COVID-19, indicating the inflammatory immune response leads to subsequent gut dysfunction [89].

Severe COVID-19 patients have experience hypoxemia and require the use of continuous positive airway pressure (CPAP) machines. Interestingly, administration of probiotic bacteria SLAB51 (a formula of nine live bacterial strains) reduced the need for CPAP [90]. Indeed, hypoxia correlates strongly with COVID-19 mortality, and a subset of patients with hypoxia remain asymptomatic for COVID-19 [91]. A recent study demonstrates that elevated fecal calprotectin (increased gut permeability) is significantly associated with nausea, vomiting, and diarrhea, and separately with hypoxemia [92]. Although there is no direct evidence of gut hypoxia in relation to COVID-19, intermittent hypoxia affects the gut and hepatic environment in type II diabetes and obstructive sleep apnea, which also potentiates inflammation and gut epithelial damage [93,94].

Fluid and electrolyte imbalance also underlie GI tract symptoms in COVID-19. Viruses such as rotavirus and SARS-CoV-2 may induce gut cells to secrete water and electrolytes through direct interactions, or through osmotic flow as a result of damaged intestinal epithelial cells and gut barrier [95].

Pneumonia increases the activity of the sympathetic nervous system [96]. In altered nervous system activities in stroke patients and pre-clinical models, there is impaired gut function, dysbiosis, and diarrhea [97,98]. Whether SARS-CoV-2 induces gut injury through these and other mechanisms remain to be elucidated.

### 4.4. Inflammatory Bowel Disease (IBD) and COVID-19

IBD is an umbrella term for a range of chronic inflammatory disorders of the GI tract that include Crohn’s disease (CD) and ulcerative colitis (UC) [99]. Initial research into patients with COVID-19 have seen many similarities in the gut when compared to patients with IBD. Patients with either IBD or COVID-19 develop inflammation associated with a dysregulated immune response in the gut, and it was therefore postulated that IBD patients may have an increased risk of developing COVID-19. A metabolomics study also showed that patients with either IBD or COVID-19 have remarkable gut dysbiosis that is associated with similar gut microbial changes, including the depletion of *F. prausnitzii*., *E. rectale*, *R. bromii* and *Lachnospiraceae*, and the overgrowth of *Enterococcus*, *E. coli* and *Shigella* [100]. These changes to the gut microbiota composition are associated with an inflammatory state in the gut and the periphery. Indeed, in a murine model of dextran sodium sulphate (DSS)-induced experimental colitis, administration of *F. prausnitzii* was able to ameliorate the development of colitis [101]. Evidence from both IBD and COVID-19 indicate the presence of gut barrier dysfunction and resultant GI symptoms such as diarrhea (and blood in stool in IBD). Many triggers, such as chemical agents (DSS) or viral infections, induce colitis in the experimental setting by directly damaging enterocytes. It is thus expected to raise the concern that IBD patients may be more susceptible to COVID-19 induced gut injury, or experience worse GI damage and symptoms. Surprisingly, many recent studies demonstrate that IBD patients have no or even low-risk to COVID-19 infection [102]. A possible reason is that these patients have routinely received anti-TNF-α treatment, and anti-TNF-α antibody is associated with lower hospitalization and less-severe COVID-19 [103]. A recent meta-analysis study has also confirmed that IBD patients received anti-TNF-α therapy have lower risk of hospitalization than those treated with steroids or mesalamine (an anti-inflammation drug to treat UC) [103].

## 5. Treatment of COVID-19 and GI Injury

### 5.1. Current COVID-19 Treatment

There is currently no cure for COVID-19 patients, and only one medication has been approved to treat COVID-19. The Food and Drug Administration (FDA) has approved intravenous administration of the anti-viral drug Remdesivir (Veklury) to treat COVID-19 in hospitalized adults and children who are aged 12 and older. Remdesivir was originally developed to treat hepatitis C, and researchers subsequently investigated its efficacy in Ebola virus infection and now extended to COVID-19. Remdesivir inhibits the RNA-dependent RNA polymerase (RdRp) in SARS-CoV-2 that is an essential enzyme for the virus to replicate and transcribe their viral RNA into the host cells [104]. However, a recent mortality trial shows that Remdesivir has limited effect on hospitalized patients with COVID-19, in terms of reducing mortality, initiation of ventilation and duration of hospital stay [105].

Molnupiravir is another antiviral drug that also targets RdRp of SARS-CoV-2, in that it interferes with viral replication and blocks viral RNA transmission. In contrast to Remdesivir that is administered by infusion, molnupiravir is delivered orally [106]. A recent phase II study shows that unvaccinated COVID-19 patients who received 800 mg molnupiravir orally twice daily for 5 days have faster viral RNA clearance than placebo controls, indicating molnupiravir is a promising candidate of COVID-19 drugs [107].

Nirmatrelvir (Paxlovid) is authorized by the FDA for COVID-19 treatment. It is a protease inhibitor, and it blocks viral protease M^PRO^ which is an essential molecule for viral replication [108]. Studies show an 89% reduction in the risk of hospitalization or death 4 weeks post-infection when Nirmatrelvir was given within 5 days of initial symptoms to patients with mild to moderate COVID-19. However, there is currently not sufficient data on clinical efficacy of Nirmatrelvir against SARS-CoV-2 variants and subvariants, such as Omicron.

Baricitinib is a medication to treat rheumatoid arthritis by reducing inflammation through inhibition of JAK1 and 2 signaling. The capacity of Baricitinib to treat COVID-19 was predicted by artificial intelligence algorithms. Studies shows that baricitinib results in a rapid decline in SARS-CoV-2 viral load and IL-6 levels in COVID-19 patients [109]. Studies also show that a baricitinib and remdesivir combination reduces recovery time and numerous serious adverse events in patients with COVID-19 [110].

Monoclonal antibodies are a class of antiviral interventions that bind to viruses and block the entry of the virus into the host cells. Spike (S) protein is the primary antigenic epitope of SARS-CoV-2, upon engaging the cell surface ACE2 receptor, and facilitates binding and fusion of the virus with the target cells. Monoclonal antibodies directly binding to the S protein can block the binding of virus to host cells in human. Some monoclonal antibodies have been granted emergency use authorization to treat patients with mild to moderate COVID-19. Studies show that a combination of casirivimab and imdevimab is able to bind all known mutants of S protein in SARS-CoV-2 and reduces viral load in experimental models of COVID-19 in animals [111]. These two antibodies have also been shown to reduce viral load in COVID-19 patients based on partial results from ongoing clinal trials [112]. Bamlanivimab and etesevimab is another combination of antibodies that decreases viral load and shorten viral clearance time in patients with mild to moderate COVID-19 [112].

Dexamethasone is a corticosteroid that is widely used to treat inflammatory disorders. Inflammation is a key pathological feature in patients with severe COVID-19 and they have significantly increased levels of inflammatory markers, including IL-1 and IL-6 [109,113]. In a UK clinical study, dexamethasone treatment reduces 28-day mortality of COVID-19 patients with ventilators by one third, and it further decreases the number of patients that received oxygen by one fifth [113].

### 5.2. Treatment for COVID-19 Induced Gut Injury

There are no effective treatments that specifically target COVID-19-induced gut injury. However, some medications have a potential role to inhibit GI symptoms and maintain intestinal microflora homeostasis in the patients with COVID-19. More than 70% residential immune cells in human are located in the GI tract to protect against microbes, indicating a strong link between the host immune system and microbiota [114]. The gut microbiota also produces vitamins, fatty acids and bile acids that can regulate the immune response. For example, studies show that lactic acid-producing bacteria stimulate the immune system to generate antiviral antibodies [115]. Probiotics also play an important role in maintaining gut microbiota composition, reducing inflammation and regulating immune response against bacterial and virus infection. Previous in vivo studies show that the genus *Lactobacillus* increases immune response against respiratory virus infection and also attenuates diarrhea [116]. The effect of probiotics, including *Lactobacillus*, have been tested in a double-blinded clinical trial (NCT04399252) in which patients take the *Lactobacillus* orally every day reduce COVID-19 symptoms [117]. *Bifidobacterium* have an anti-inflammatory role in regulating T cell infiltration and the secretion of proinflammatory cytokines, including TNF-α and IL-10 [118]. A recent study shows that COVID-19 patients have a reduction or even depletion of *Bifidobacterium* in their guts [119]. Studies also show that COVID-19 severity is associated with decreased *Bifidobacterium* and *Faecalibacterium* [119]. Therefore, probiotics potentially protect against COVID-19-induced microbiota changes (Table 1).

SARS-CoV-2 binds to the human ACE2 receptor to enter host cells, and hACE2 expression is significantly increased in the gut of COVID-19 patients [120]. Therefore, prevention of interactions between SARS-CoV-2 receptor-binding domain (RBD) and ACE2 may be a strategy to inhibit COVID-19. ACE2 inhibitor was thought of as an important drug to inhibit SARS-CoV-2 binding. However, ACE2 is a key molecule in maintaining the normal function of many organs, including lungs, heart, kidneys and GI tracts, while maintaining microbial ecology [76,121]. Therefore, direct inhibition of ACE2 in the body may cause pathogenesis of other diseases among COVID-19 patients. Injection of hACE2 recombinant protein is a promising approach to block the virus binding to ACE2 on the host cells in gut. Previous studies have shown that soluble human ACE2 recombinant protein reduces SARS-CoV-2 infection in capillary and kidney organoids [122]. Intravenous delivery of hACE2 recombinant protein twice daily has been used in a phase II clinical trial (NCT04335136).

Dietary nutrients are important to restore the function of digestion in the GI tract and maintain mucosal immunity in COVID-19 patients with injury in the GI tract. Omega-3 fatty acid and docosahexaenoic acid (DHA) can inactivate the enveloped virus by modulating the host lipid condition for viral replication, indicating their role in the reduction of severity and improving recovery of COVID-19 in the patients [123]. Carbohydrates and dietary fiber are also involved in the regulation of immune responses in human in that taking low-carbohydrate and high-fibrer food may affect COVID-19 with GI injury [124]. Vitamins, folate and iron play an important role in boosting the immune system and deficiencies of these nutrients reduce resistance against infection, such as SARS-CoV-2. Vitamin C has been used to treat severe COVID-19 patients in a clinical trial (NCT04264533), but it was terminated due to a decreased sample size in the second outbreak of COVID-19 in China. Many other clinical trials (NCT04401150, NCT04357782, NCT04344184, NCT04682574, NCT04710329) on using vitamin C to treat COVID-19 are currently under recruitment or in the process of data analysis [125], and the results will be available to the public soon. There is also preliminary evidence for the use of zinc supplementation to alleviate COVID-19 induced gut injury, especially to treat diarrhea [126]. In addition, in patients with PACS, nutritional modulation through a combination of increased fiber (formulated under NBT-NM108) and an investigational new drug (IND155864) reverted dysbiosis and reduced GI tract symptoms including loss of appetite and nausea [127].

Other therapies are also being explored for COVID-19. A selection of herbal medicine is not only safe but show a degree of efficacy to reduce COVID-19 symptoms, and the use of these herbs could be further discussed for clinical study [128]. A new strategy to treat COVID-19 aims to improve gut barrier function, such as larazotide, an antagonist of zonulin, increases gut permeability. A pilot clinical trial in pediatric COVID-19 indicates that children who received larazotide showed significantly faster improvement in GI symptoms and a shorter length of stay in hospital [129]. Given the increasing evidence that the gut microbiota and environment play an important role in COVID-19 induced GI symptoms, other strategies such as symbiotics and postbiotics could also present as valuable treatment, though these remain to be investigated in detail. Excitingly, there is evidence of using intestinal cells (Caco-2 cells) and organoids to test the characteristics of SARS-CoV-2 variants, and for the effects of the gut microbiota, thereby assisting in quicker translation of therapies into clinical practice [130,131].

SARS-CoV-2 infects the respiratory system, and the virus translocates to gastrointestinal (GI) tract. This leads to immune cell recruitments in the gut, including neutrophils, macrophages, dendritic cells and lymphocytes. The infection also results in these immune cells to secrete cytokines and microbiota changes in the GI tract.

SARS-CoV-2 directly infects gut epithelial cells, and results in impaired gut barrier function and microbial translocation. This induces inflammation (e.g., by cytokine storm) that potentiates dysregulation of gut microbiota, metabolites, electrolytes, and gut barrier functions. The infection of SARS-CoV-2 into the host cells is associated with neutrophilia and lymphopenia, and impairs the host anti-viral immune response that generates a positive feedback loop to promote further infection and damage. These processes lead to damage of the GI tract and associated symptoms such as diarrhea. IFNs: interferons. IL: interleukin. CRP: C-reactive protein. LPS: lipopolysaccharide. TNF: tumour necrosis factor.

## 6. Conclusions

SARS-CoV-2 predominately targets the respiratory system, but symptoms in the GI tract is also very common in COVID-19 patients. In this review, we summarized clinical features of COVID-19-induced gut injury. Inflammatory cells and their secreted cytokines and microbiota are important in COVID-19-induced gut damage. GI symptoms in COVID-19 patients should be considered and paid attention to in the diagnosis and treatment. Therapeutics that target to the GI tract, such as probiotics, ACE2 inhibitors and dietary nutrients, are promising options for COVID-19-inducd gut injury.

## Figures and Tables

**Table 1:** Current probiotics clinical trials related to gut injury of COVID-19 patients.

Sponsor	Targets	Clinical Trial Identifier	Status
I.M. Sechenov First Moscow State Medical University, Moscow, Russia	*Lactobacillus rhamnosus*; *Bifidobacterium bifidum*; *B. longum* subsp. *Infantis*; *Bifidobacterium longum*	NCT04854941	Completed
Centre de recherche du Centre hospitalier universitaire de Sherbrooke, Sherbrooke, Canada	2 strains (unknown)	NCT05080244	Recruiting
King’s College Hospital NHS Trust	*Bifidobacterium*; *Lactobacillus*	NCT04877704	Not yet recruiting
Medi Pharma Vision	Probiotics (unknown)	NCT05474144	Completed
Bioithas SL, London, UK	Probiotics (unknown)	NCT04390477	Completed
Centre hospitalier de l’Université de Montréal (CHUM), Montréal, Canada	Probiotics (unknown)	NCT04458519	Completed
Nordic Biotic Sp. Z o.o., Warsaw, Poland	Probiotics (unknown)	NCT04907877	Recruiting
Université de Sherbrooke, Sherbrooke, Canada	Probiotics (unknown)	NCT05195151	Not yet recruiting
Biosearch S.A., Granada, Spain	*Lactobacillus Coryniformis* K8	NCT04366180	Recruiting
ProbiSearch SL, Madrid, Spain	*Lactobacillus salivarius* + Vitamin D + Zinc	NCT04937556	Completed
Vanderbilt University Medical Center, Nashville, USA	Magnesium Citrate + probiotics (unknown)	NCT04941703	Recruiting
King Edward Medical University, Punjab, Pakistan	*Streptococcus salivarius* K12	NCT05043376	Completed
Universidade de Passo Fundo, Passo Fundo, Brazil	*Streptococcus salivarius* K12; *Lactobacillus brevis* CD2	NCT05175833	Completed
Indonesia University, Jakarta, Indonesia	Probiotics (unknown) + Vitamin D	NCT04979065	Recruiting
Biosearch S.A. Granada, Spain	*Lactobacillus*	NCT04756466	Active, not recruiting
Chinese University of Hong Kong, Hong Kong, China	Microbiome immunity formula (unknown)	NCT04950803	Recruiting
Rutgers, The State University of New Jersey, New Brunswick, USA	A combination of live microbials (unknown)	NCT04847349	Completed
Medical University of Graz, Graz, Austria	Omni-Biotic Pro Vi 5 (unknown probiotics)	NCT04813718	Active, not recruiting
AB Biotek, Barcelona, Spain	*Saccharomyces cerevisiae*	NCT04798677	Completed
Medical College of Wisconsin, Milwaukee, USA	*Lactobacillus Plantarum*	NCT05227170	Recruiting
Universidad Complutense de Madrid, Madrid, Spain	*Ligilactobacillus salivarius*	NCT04922918	Recruiting
AB Biotics, SA, Mexico city, Mexico	*Lactobacillus plantarum*, *Lactobacillus plantarum*, *Lactobacillus plantarum*, *Pediococcus acidilactici*	NCT04517422	Completed
Duke University, Durham, USA	*Lactobacillus rhamnosus*	NCT04399252	Completed

## Data Availability

Not applicable.
